# MicroRNA‐7‐5p induces cell growth inhibition, cell cycle arrest and apoptosis by targeting PAK2 in non‐small cell lung cancer

**DOI:** 10.1002/2211-5463.12738

**Published:** 2019-10-24

**Authors:** Qin Li, Xingping Wu, Lin Guo, Jiaxin Shi, Jiashu Li

**Affiliations:** ^1^ Department of Respiration Xuzhou Medical University Affiliated Hospital of Lianyungang China

**Keywords:** cell proliferation, miR‐7‐5p, non‐small‐cell lung cancer, PAK2

## Abstract

MicroRNAs (miR) are known to be critical regulators in tumor progression. miR‐7‐5p was reported to be involved in several cancers, including glioblastoma, cervical cancer, and melanoma, but its prognostic value and biological function in non‐small‐cell lung cancer (NSCLC) remain unclear. In this study, using quantitative real‐time PCR analysis, we found that miR‐7‐5p was significantly downregulated in NSCLC tissues and cell lines. Lower miR‐7‐5p expression was associated with tumor–node–metastasis stage and tumor size by chi‐squared test. Deceased miR‐7‐5p expression was associated with a worse prognosis in patients with NSCLC using Kaplan–Meier survival analysis and multivariate Cox regression analysis. Experiments in NSCLC cell lines (A549 and H1299) demonstrated that upregulation of miR‐7‐5p significantly suppressed cell proliferation, but induced cell cycle G0/G1 phase arrest and apoptosis using Cell Counting Kit‐8, colony formation, and flow cytometry analysis. Through loss‐of‐function assays, we further demonstrated that downregulation of miR‐7‐5p promoted cell proliferation and cell cycle G1/S transition, but decreased cell apoptosis in SPC‐A1 cells. Furthermore, P21‐activated kinase 2 (PAK2) was predicted and confirmed as a direct target gene of miR‐7‐5p in NSCLC cells by luciferase reporter assay. In addition, we found PAK2 overexpression could partially reverse the effects of miR‐7‐5p on cell proliferation, cell cycle distribution, and apoptosis. We thus concluded that lower expression of miR‐7‐5p was associated with poor prognosis and NSCLC progression by directly targeting PAK2.

**Abbreviations**
CCK‐8: Cell Counting Kit‐8
NSCLC: non‐small‐cell lung cancer
OS: overall survival
PAK2: P21‐activated kinase 2

According to the National Central Cancer Registry of China for 2015, lung cancer remains the leading cause of cancer‐related death in males and females in China 1. Non‐small‐cell lung cancer (NSCLC) represents ~ 85–90% of all cases of lung cancer 2. Rapid advances in the treatment of NSCLC have led to the understanding of the disease pathology, the molecular mechanism of cancer metastasis, management, and prognosis 3. Unfortunately, most NSCLC patients have tumor progression despite the use of surgical resection, chemoradiotherapy, EGFR‐tyrosine kinase inhibitors (TKI), and ALK inhibitors 4. Hence, continued investigation on new therapeutics and prognostic indicators is required to improve the outcomes in NSCLC.

MicroRNAs (miRNAs) are a class of endogenous, small (18–24 nucleotides), noncoding RNA species that regulate gene expression through recognizing and directly binding to a complementary site(s) in the 3′‐UTR of a target mRNA 5. Recently, more than 3000 miRNAs are annotated in the human genome and one miRNA can regulate hundreds (or thousands) of different mRNAs 6. A bunch of miRNAs has been shown to play an important role in various biological processes, including differentiation, apoptosis, and modulation of cell cycle 7. In the past two decades, deregulation of miRNAs is found to be associated with cancerous transformation and progression 8. These tiny miRNAs function as ‘oncomiR’ or tumor suppressor in multiple types of human malignancies 9. Recently, miR‐7‐5p has been reported to be strongly underexpressed in breast cancer 10, melanoma 11, glioblastoma 12, and hepatocellular carcinoma 13, which indicated that miR‐7‐5p displayed antitumor activity. Mechanically, miR‐7‐5p acts to downregulate the transcription and translational level of direct or indirect tumor promoter to suppress the proliferative and aggressive malignant phenotypes 10, 11, 12, 13. However, its clinical significance and biological function have not yet been explored in NSCLC. The p21‐activated kinases (PAKs) are a family of serine/threonine kinases that play essential roles downstream of Cdc42, Rac1, and Rho in multiple cellular processes 14. The PAK family constitutes two distinct subgroups: subgroup 1, consisting of PAKs 1–3, and subgroup 2, consisting of PAKs 4–6 15. It has been shown that PAK2 regulates actin cytoskeleton remodeling, motility, differentiation, and attachment in numerous cellular contexts, including cancer cells 14, 16, 17. Similar expression profiles of PAK2 have been found in ovarian cancer cell lines and normal ovarian epithelial cells, and overexpression of PAK2 has been reported in melanoma cells 18, 19. PAK2 deficiency strongly inhibited the migration and invasion of ovarian cancer cells, but had no effect on cell viability and apoptosis 18. Higher level of PAK2 and pSer20PAK2 was significantly correlated with poor disease prognosis and unfavorable clinicopathologic features in gastric cancer 20. Moreover, previous studies revealed that other PAK family members such as PAK4 could predict tumor progression and prognostic outcome in patients with NSCLC 21. Based on these evidences, we made a reasonable hypothesis that PAK2 might be an oncogene in NSCLC cells.

The current study investigated the clinicopathological significance and prognosis of miR‐7‐5p as well as its functional role in NSCLC. TargetScan database and luciferase reporter assay were performed to predict and validate whether PAK2 was a direct target of miR‐7‐5p in NSCLC cells. We further explored whether PAK2 was involved in miR‐7‐5p‐mediated cell behaviors. These results may provide a promising prognostic biomarker and a potential therapeutic target for treating NSCLC.

## Materials and methods

### Patients and clinical tissues

A total of 85 paired fresh tumor tissues and matched adjacent noncancerous tissues were collected from NSCLC patients between 2017 and 2018 in the Xuzhou Medical University Affiliated Hospital of Lianyungang (Jiangsu, China). None of the NSCLC patients received radiotherapy or chemotherapy treatment, and all the patients signed informed consent before surgical resection. Resected lung tissues were immediately immersed in liquid nitrogen until further experiments. The clinical characteristics, including gender, age, and smoking history, were recorded and summarized in Table 1. The survival time was calculated from the date of resection and followed up for 5 years for overall survival (OS) analysis. This study was in accordance with Helsinki Declaration and approved by the Institutional Ethics Committee of Xuzhou Medical University Affiliated Hospital of Lianyungang.

**Table 1:** Correlations of the miR‐7‐5p with clinicopathological features in NSCLC patients. *P* < 0.05 are shown in bold.

Features	All cases	miR‐7‐5p expression		*P* value (chi‐squared test)
		Low	High	
Total	85	46	39	
Gender				
Male	51	28	23	0.846
Female	34	18	16	
Age (years)				
< 60	37	19	18	0.742
≥ 60	48	27	21	
Tumor–node–metastasis stage				
I–II	37	15	22	**0.004**
III–IV	48	31	17	
Tumor size (cm)				
< 4	34	14	20	**0.007**
≥ 4	51	32	19	
Lymph node metastasis				
Negative	36	17	19	0.115
Positive	49	29	20	
Differentiation				
Good/moderate	53	27	26	0.311
Poor	32	19	13	
Smoking history				
No	33	15	18	0.109
Yes	52	31	21	

### Cell culture and transfection

Human lung epithelial cell EBAS‐2B and four NSCLC cell lines (A549, SPC‐A1, 95D, and H1299) were purchased from the American Type Culture Collection (Manassas, VA, USA). All cell lines were cultured in Dulbecco’s modified Eagle’s medium (Gibco, Carlsbad, CA, USA) supplemented with 10% FBS (Gibco) at 37 °C in an atmosphere of 5% CO_2_.

Before transfection, A549, H1299, and SPC‐A1 cells at a density of 1 × 10^5^ cells per well were seeded into six‐well plates and incubated overnight. The miR‐7‐5p mimic (mimic) and its negative control (miR‐NC) were synthesized by RiboBio Co., Ltd. (Guangzhou, China). The miR‐7‐5p inhibitor and corresponding miR‐NC were also provided by RiboBio Co., Ltd. The recombinant pcDNA3.1/PAK2 plasmid was constructed by OriGene Technologies, Inc. (Beijing, China). The empty pcDNA3.1 vector was used as a control. All cell transfections were lasted for 48 h and performed using Lipofectamine® 2000 (Invitrogen, Carlsbad, CA, USA; Thermo Fisher Scientific, Inc., Waltham, MA, USA) with 50 nm miRNA or 1 µg PAK2 plasmid according to the manufacturer's protocol.

### Quantitative real‐time PCR

Total RNA was extracted using TRIzol® Reagent (Invitrogen) according to the manufacturer's protocol. The first‐strand cDNA was synthesized using Moloney murine leukemia virus reverse transcriptase (TAKARA, Dalian, China). Quantitative real‐time PCR assays were performed on LightCycler® 480 Real‐time PCR System by LightCycler® 480 SYBR Mix (Roche Diagnostics Ltd., Shanghai, China). The expression levels of mature miR‐7‐5p and PAK2 was normalized to the expression of U6 and GAPDH, respectively, using the 2^−ΔΔCT^ method. The primers used are listed as follows: miR‐7‐5p forward, 5′‑AAAACTGCTGCCAAAACCAC‑3′ and reverse, 5′‑GCTGCATTTTACAGCGACCAA‑3′; U6 forward, 5′‑CTCGCTTCGGCAGCACATATACT‑3′ and reverse, 5′‑ACGCTTCACGAATTTGCGTGTC‑3′; PAK2 forward, 5′‑TCTTCCTCCCCCAGGGTTG‑3′ and reverse, AATCGAGCCCACTGTTCTGG; GAPDH forward, 5′‑CGGAGTCAACGGATTTGGTCGTAT‑3′ and reverse, 5′‑AGCCTTCTCCATGGTGGTGAAGAC‑3′.

### CCK‐8 assay

After cell transfection, cells were seeded into a 96‐well plate at a density of 3 × 10^3^ cells per well and cultured for overnight. Subsequently, 10 μL of CCK‐8 (Dojindo, Kumamoto, Japan) was added into each well at indicated time points (24, 48, 72, and 96 h, respectively). Following incubation of cells for another 2 h, the optical density values in each well were measured at 450 nm using a microplate reader.

### Colony formation assay

After cell transfection, cells were seeded into six‐well plates (500 cells per well) and incubated at 37 °C for 10 days. At day 10, each well was washed with PBS for three times. The naturally formed colonies were fixed by methanol for 30 min stained with 0.5% crystal violet at the room temperature for 30 min. Visible cell colonies (a group of > 50 cells) were observed and counted in five random fields.

### Cell cycle and apoptosis assays

After cell transfection, cells were detached with trypsin and rinsed with PBS two times. For cell cycle distribution detection, cells were fixed with cool 70% ethanol at −20 °C overnight and stained with a mixture of 50 mg·mL^−1^ PI (Sigma‐Aldrich, St. Louis, MO, USA) and 25 mg·mL^−1^ RNase A for 30 min in the dark. For cell apoptosis detection, cells were collected and incubated with 5 μL Annexin V‐FITC and 5 μL PI (Sigma‐Aldrich) for 15 min in the dark. Analyses of cell cycle and cellular apoptosis were performed using a flow cytometer, FACSCalibur (BD Biosciences, San Jose, CA, USA).

### Dual‐luciferase reporter assay

The potential binding sites of miR‐7‐5p with PAK2 were predicted by TargetScan database (http://www.targetscan.org/vert_71/). The human wild‐type (WT) PAK2 was amplified and cloned into luciferase reporter vector psiCHECKTM‐2 (Promega, Madison, WI, USA) to generate WT PAK2 plasmid. Meanwhile, PAK2 gene mutant 3′‐UTR recombinant plasmid (MUT PAK2) was generated using the TaKaRa MutanBEST Kit (TaKaRa, Beijing, China), which generated a mutation of 7 bps from GUCUUCC to ACUCCUU in the predicted miR‐7‐5p target binding site. Next, A549 or H1299 cells were seeded in 24‐well plates and cotransfected with 150 ng of WT or MUT PAK2 plasmid together with 50 nm miR‐7‐5p mimic or miR‐NC using Lipofectamine 2000. At 48 h after cotransfection, the activities of Firefly and Renilla luciferase were measured and relative luciferase activity was calculated with the Dual‐Luciferase Reporter Assay Kit (Promega).

### Western blotting

Total protein was extracted using radioimmunoprecipitation assay buffer (Beyotime, Beijing, China). Equal amounts of proteins were separated by and then transferred onto polyvinylidene fluoride (Millipore, Billerica, MA, USA) membranes. The membrane was blocked with 5% nonfat dried milk powder in TBST and incubated with primary antibodies against PAK2 and GAPDH at 4 °C overnight, followed by incubation with horseradish peroxidase‐conjugated secondary antibodies for 2 h at room temperature. The protein bands were visualized using an enhanced chemiluminescence kit (Amersham Biosciences, Piscataway, NJ, USA) with GAPDH used as an internal control.

### Statistical analysis

All statistical analysis was performed using the software package spss version 21.0 (Chicago, IL, USA). The data are shown as the means ± SD. Relationships between miR‐7‐5p expression and the clinicopathological features were analyzed via the chi‐squared test. The survival analysis was performed using the Kaplan–Meier method, and differences in survival curves were evaluated by log‐rank test. Multivariate Cox regression models were used to assess factors associated with OS in NSCLC. All *in vitro* experiments were carried out in triplicate and were repeated three times. The data were statistically analyzed by Student’s *t*‐test for comparing two groups and one‐way analysis of variance for comparing multiple groups. All *P*‐values < 0.05 were considered statistically significant.

## Results

### Relationship between deceased miR‐7‐5p expression and clinicopathological features of NSCLC patients

**Figure 1:**
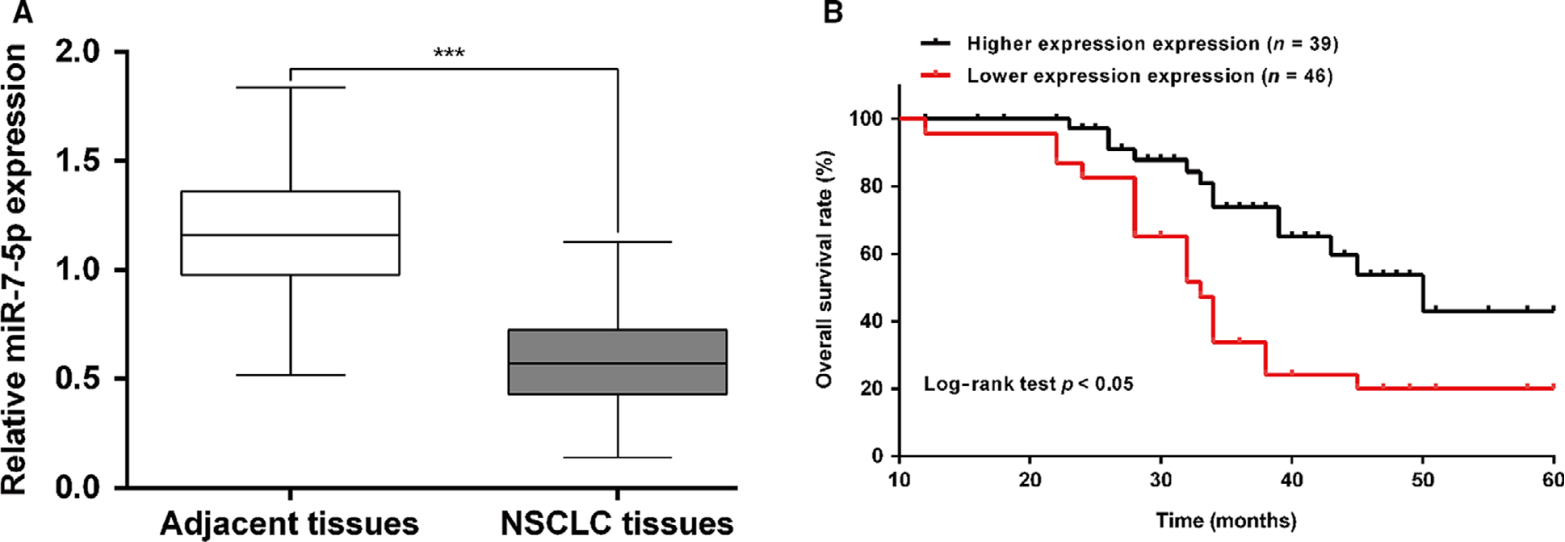
The expression of miR‐7‐5p and its association with survival prognosis in patients with NSCLC. (A) Expression levels of miR‐7‐5p were evaluated by quantitative real‐time PCR in tissue samples collected from NSCLC patients. Data are shown as the means ± SD of three independent experiments. The data were statistically analyzed by Student’s *t*‐test. (B) Kaplan–Meier survival curves and log‐rank tests were used to compare OS of NSCLC patients with high and low expression levels of miR‐7‐5p. ***P < 0.001, compared with adjacent tissues.

To explore the potential role of miR‐7‐5p in NSCLC pathogenesis, the expression of mature miR‐7‐5p was analyzed in 85 NSCLC tissue samples and their adjacent normal lung tissues by quantitative real‐time PCR. As shown in Fig. 1A, the expression of miR‐7‐5p was significantly reduced in NSCLC patients compared with that in adjacent normal tissues (*P* < 0.001). According to the median values of miR‐7‐5p expression, 85 NSCLC patients were divided into high miR‐7‐5p expression group (*n* = 39) and low miR‐7‐5p expression group (*n* = 46). Using chi‐squared test, we assessed the association between miR‐7‐5p expression and clinical characteristics. As depicted in Table 1, decreased miR‐7‐5p expression was significantly correlated with tumor–node–metastasis stage (*P* = 0.004) and tumor size (*P* = 0.007). Conversely, no relationship was found between miR‐7‐5p expression and gender, age, lymph node metastasis, differentiation, and smoking history (all *P* > 0.05).

### Deceased miR‐7‐5p expression was associated with a worse prognosis in patients with NSCLC

We subsequently analyzed the relationship between miR‐7‐5p expression and OS in 85 patients with NSCLC using Kaplan–Meier survival analysis. As shown in Fig. 1B, we found lower expression of miR‐7‐5p was significantly associated with poorer OS (log‐rank *P* < 0.05). Moreover, multivariate Cox regression was conducted to analyze the potential factors independently correlated with OS. The results indicated that miR‐7‐5p was an independent prognostic factor for NSCLC patients (Table 2, hazard ratio = 2.03, 95% confidence interval = 1.75–2.83, *P* = 0.036).

**Table 2:** Multivariate Cox regression analysis of factors associated with OS in NSCLC. *P* < 0.05 are shown in bold.

Characteristics	Multivariate analysis	
	Hazard ratio (95% confidence interval)	*P* value
Gender (male vs. female)	2.34 (1.96–3.19)	0.613
Age (< 60 vs. ≥ 60)	1.56 (1.03–1.98)	0.462
Tumor–node–metastasis stage (I–II vs. III–IV)	2.13 (1.94–2.59)	**0.012**
Tumor size (< 4 vs. ≥ 4 cm)	2.03 (1.64–2.84)	0.086
Lymph node metastasis (negative vs. positive)	1.28 (0.99–1.67)	0.349
Differentiation (good/moderate vs. Poor)	2.06 (1.85–2.74)	0.420
Smoking history (no vs. yes)	1.37 (0.94–1.96)	0.316
miR‐7‐5p expression (low vs. High)	2.03 (1.75–2.83)	**0.036**

### Effects of miR‐7‐5p on cell proliferation, cell cycle progression, and apoptosis in NSCLC cells

**Figure 2:**
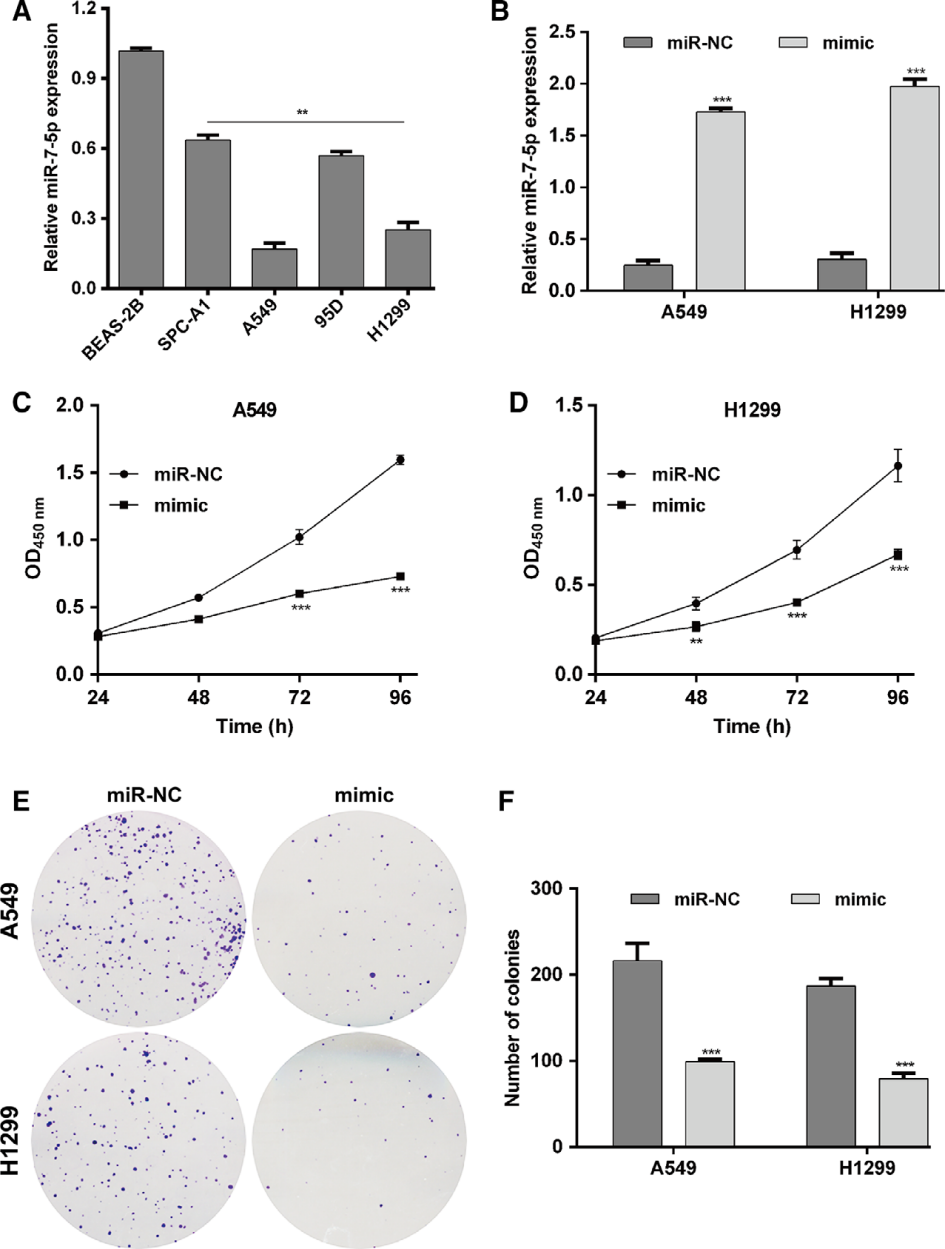
Upregulation of miR‐7‐5p suppressed cell proliferation in NSCLC cells. (A) Quantitative real‐time PCR analysis was used to detect the miR‐7‐5p expression in NSCLC cell lines (A549, SPC‐A1, 95D, and H1299) and the lung epithelial cell EBAS‐2B (**P < 0.01 vs. EBAS‐2B). (B) Expression of miR‐7‐5p was analyzed by quantitative real‐time PCR after transfection with miR‐7‐5p mimic (mimic) or miR‐NC in A549 or H1299 cells, respectively. CCK‐8 assay was performed to detect the proliferation of (C) A549 and (D) H1299 cells transfected with mimic or miR‐NC, respectively. (E) Colony formation ability was assessed using colony formation assay in A549 and H1299 cells; representative images (left panel) and statistical analysis (right panel) are shown. The data were statistically analyzed by Student’s *t*‐test for comparing two groups and one‐way analysis of variance for comparing multiple groups. Data are shown as the means ± SD of three independent experiments. **P < 0.01, ***P < 0.001 vs. miR‐NC.

**Figure 3:**
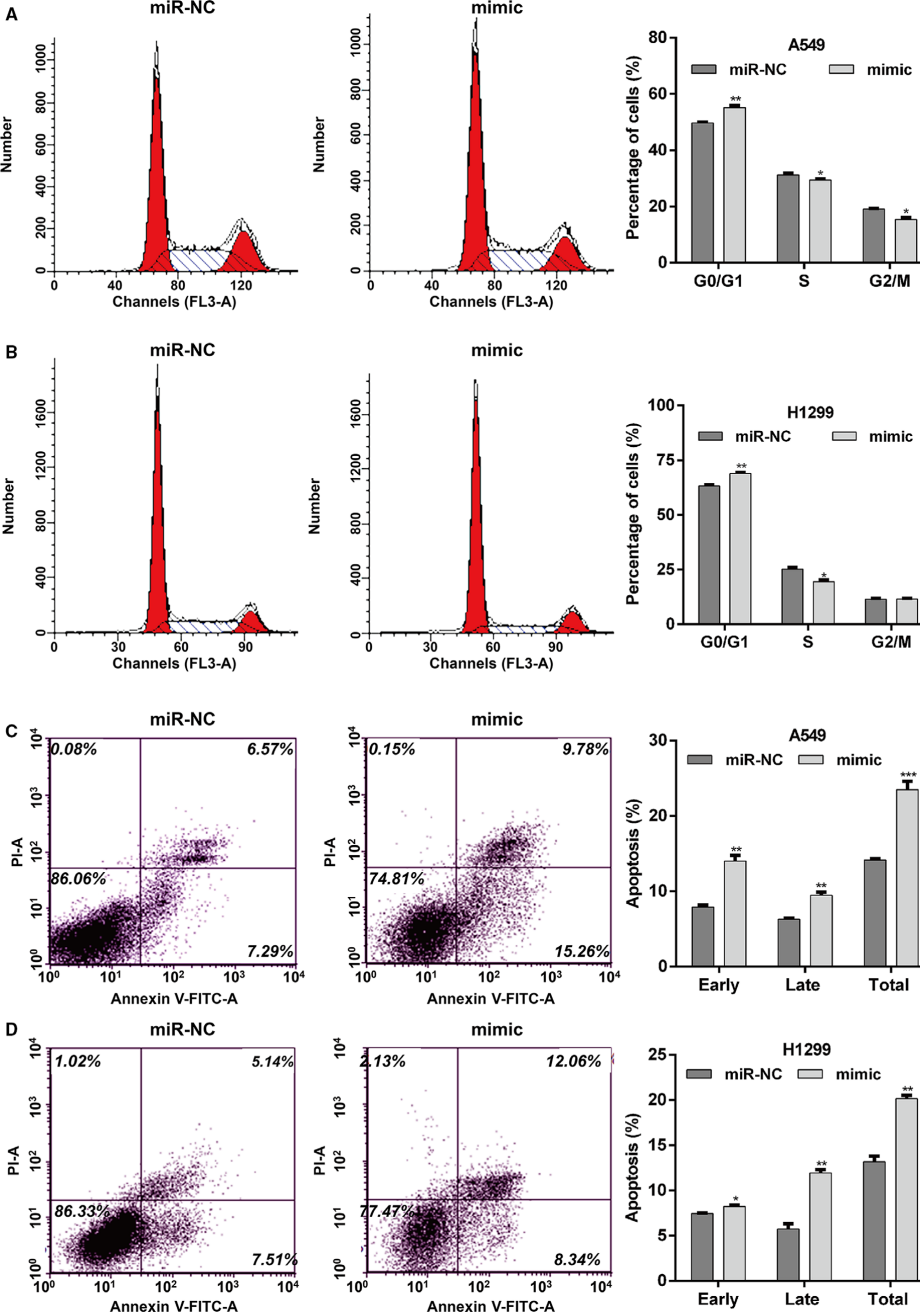
Upregulation of miR‐7‐5p induced cell cycle arrest and apoptosis in NSCLC cells. DNA content was analyzed in (A) A549 and (B) H1299 cells transfected with mimic or miR‐NC using flow cytometry, and the percentage of cells in the G0/G1, S, and G2/M phases of the cell cycle was calculated. Apoptosis rate of (C) A549 and (D) H1299 cells following transfection with mimic or miR‐NC was examined by flow cytometric analysis. The data were statistically analyzed by one‐way analysis of variance for comparing multiple groups. Data are shown as the means ± SD of three independent experiments. *P < 0.05, **P < 0.01, ***P < 0.001 vs. miR‐NC.

**Figure 4:**
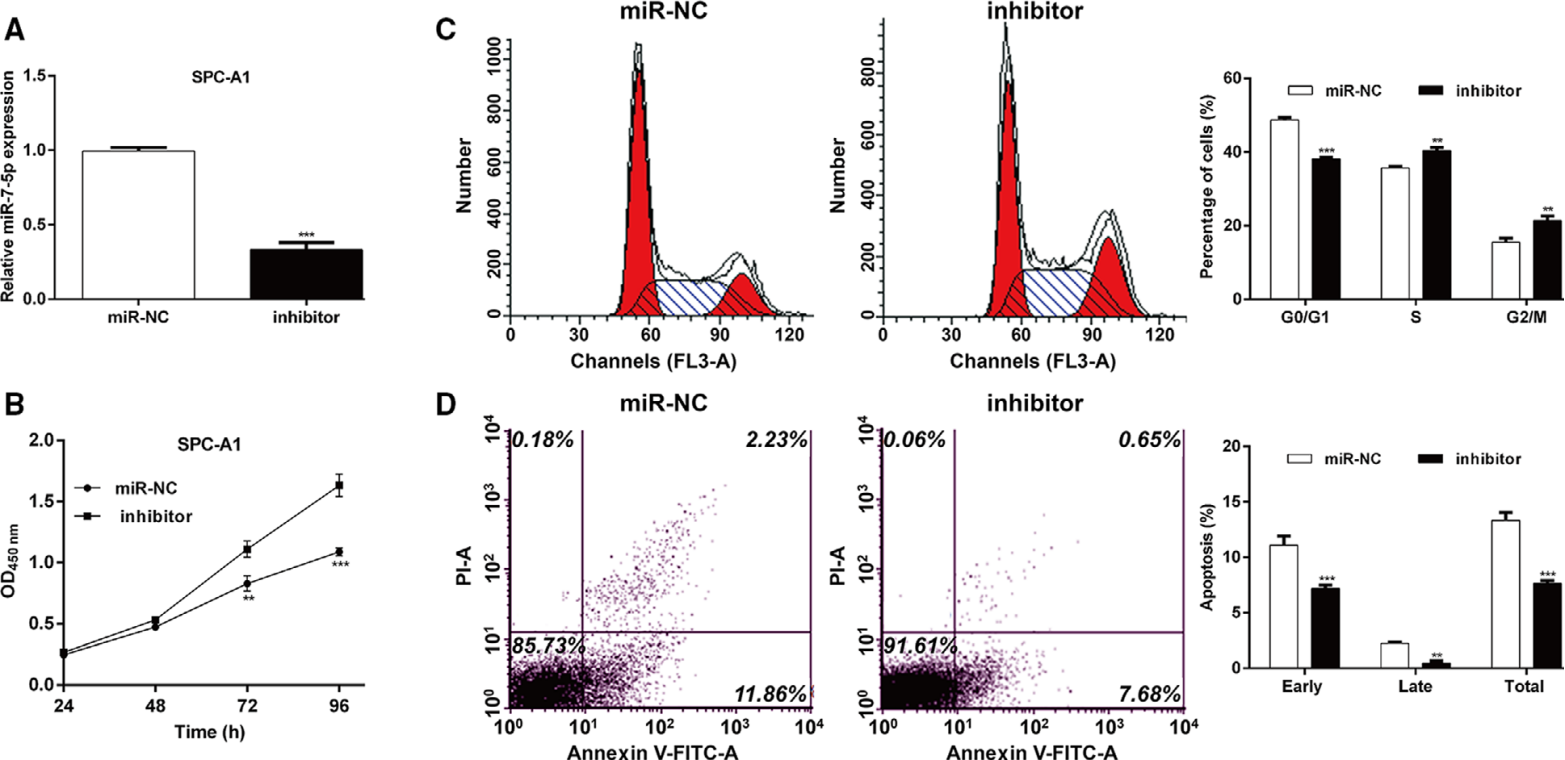
Downregulation of miR‐7‐5p promoted cell proliferation, cell cycle progression, and decreased apoptosis. SPC‐A1 cells were transfected with miR‐7‐5p inhibitor (inhibitor) or miR‐NC. (A) Expression of miR‐7‐5p was analyzed by quantitative real‐time PCR. (B) CCK‐8 assay was performed to detect the proliferation of SPC‐A1 cells. (C) Cell cycle distribution and (D) apoptosis were analyzed using flow cytometry analysis. The data were statistically analyzed by Student’s *t*‐test for comparing two groups. Data are shown as the means ± SD of three independent experiments. **P < 0.01, ***P < 0.001 vs. miR‐NC.

To investigate the functional role of miR‐7‐5p in NSCLC *in vitro*, the expression of miR‐7‐5p was determined in several NSCLC cell lines using quantitative real‐time PCR. As shown in Fig. 2A, miR‐7‐5p was significantly downregulated in four NSCLC cell lines compared with lung epithelial cell EBAS‐2B (*P* < 0.01). Among the investigated NSCLC cell lines, A549 and H1299 cells exhibited relatively lower miR‐7‐5p expression, while SPC‐A1 cells presented relatively higher miR‐7‐5p expression, and thus were selected as cell models to perform gain‐of‐function and loss‐of‐function assays. We first augmented the expression levels of miR‐7‐5p by transfecting A549 or H1299 cells with mimic, followed by quantitative real‐time PCR analysis. As shown in Fig. 2B, mimic transfection remarkably elevated the expression of miR‐7‐5p in both A549 and H1299 cells (*P* < 0.001). The CCK‐8 assay results revealed cell growth rate was repressed in the A549 (Fig. 2C, *P* < 0.001) and H1299 cells (Fig. 2D, *P* < 0.01, *P* < 0.001) with mimic transfection compared with miR‐NC transfection. Moreover, colony formation assay (Fig. 2E, *P* < 0.001) demonstrated that miR‐7‐5p overexpression obviously reduced the number of colonies in A549 and H1299 cells. Then, we examined whether miR‐7‐5p overexpression affected cell cycle progression of induced apoptosis. The results demonstrated that miR‐7‐5p overexpression induced G0/G1 cell cycle arrest, as reflected by an increased percentage of cells in the G0/G1 phase (*P* < 0.01) and a decreased percentage of cells in the S phase (*P* < 0.05) and G2/M (*P* < 0.05) in mimic transfected A549 cells (Fig. 3A). Similarly, the percentage of cells in G0/G1 (*P* < 0.01) was significantly elevated, while that in S phase (*P* < 0.05) was accordingly reduced in H1299 cells after miR‐7‐5p overexpression (Fig. 3B). Additionally, we further found the apoptotic rate, including early and late apoptosis of A549 (Fig. 3C, *P* < 0.01, *P* < 0.001) and H1299 cells (Fig. 3D, *P* < 0.05, *P* < 0.01), was obviously increased after mimic transfection, in comparison with miR‐NC transfection. Furthermore, SPC‐A1 cells were transfected with inhibitor to achieve miR‐7‐5p knockdown, as demonstrated by quantitative real‐time PCR (Fig. 4A, *P* < 0.001). Opposite with gain‐of‐function assay, downregulation of miR‐7‐5p significantly promoted cell proliferation (Fig. 4B, *P* < 0.01, *P* < 0.001) and cell cycle G1/S transition (Fig. 4C, *P* < 0.01, *P* < 0.001), but decreased cell apoptosis (Fig. 4D, *P* < 0.01, *P* < 0.001) in SPC‐A1 cells, as determined by CCK‐8 assay and flow cytometry analysis. Collectively, these data indicate that miR‐7‐5p could suppress cell proliferation and induced G0/G1 phase arrest and apoptosis in NSCLC cells.

### PAK2 was the direct target of miR‐7‐5p in NSCLC

**Figure 5:**
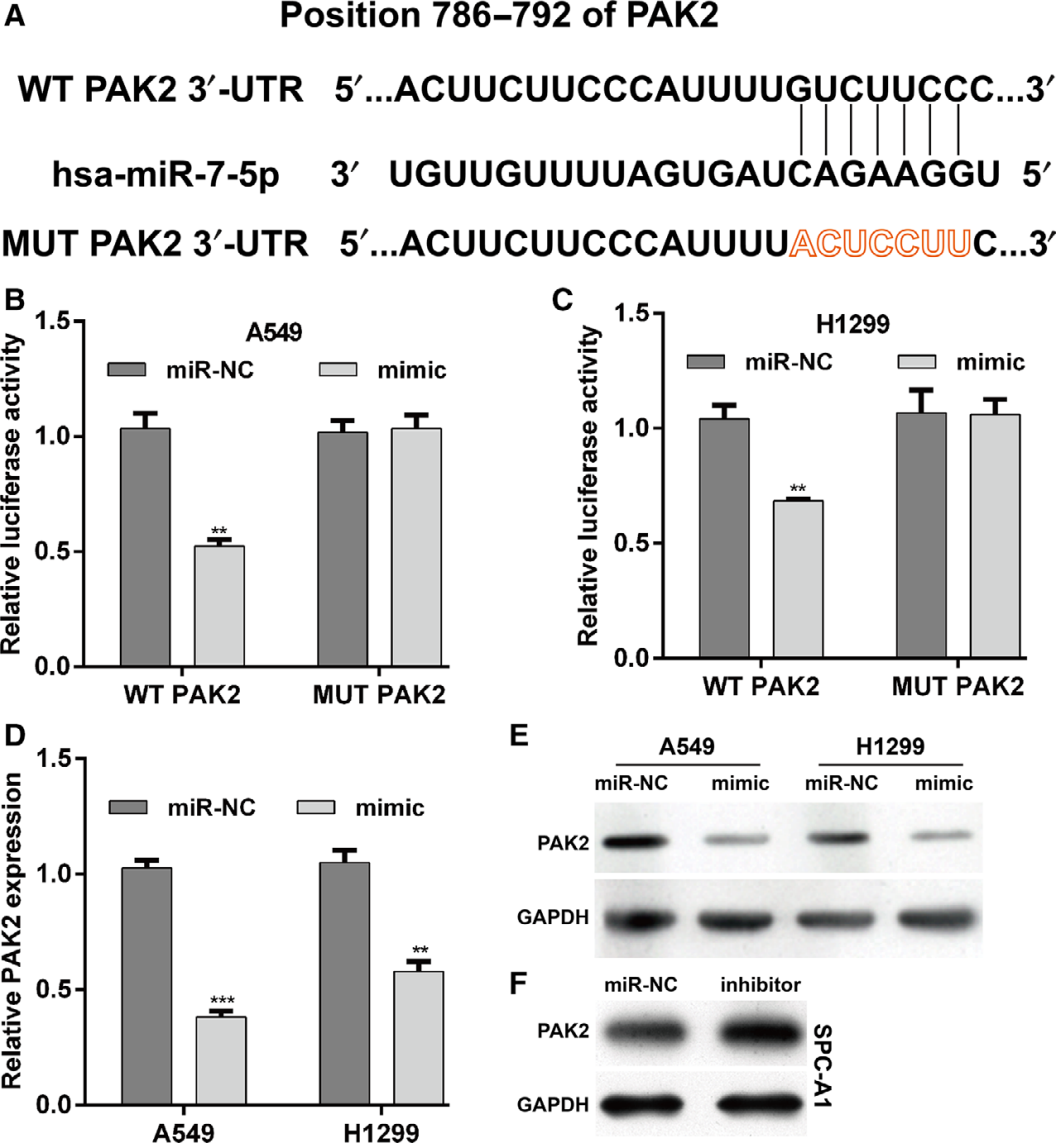
MiR‐7‐5p directly targets PAK2 in NSCLC cells. (A) Schematic representation shows the predicted target sites of miR‐7‐5p at the 3′‐UTR of PAK2. A mutation in the seed matches is referred to as PAK2‐MUT. Luciferase reporter assay was performed in (B) A549 and (C) H1299 cells. The (D) mRNA and (E) protein expression of PAK2 was analyzed in A549 and H1299 cells containing miR‐7‐5p mimic or miR‐NC. (F) The protein expression of PAK2 was measured in SPC‐A1 cells after transfected with inhibitor or miR‐NC. The data were statistically analyzed by one‐way analysis of variance for comparing multiple groups. Data are shown as the means ± SD of three independent experiments. **P < 0.01, ***P < 0.001 vs. miR‐NC.

To elucidate the molecular mechanism underlying the effects of miR‐7‐5p on NSCLC cells, the potential target genes of miR‐7‐5p were predicted. As shown in Fig. 5A, PAK2 was identified as a potential target gene of miR‐7‐5p, as the 3’‐UTR region of human PAK2 contained a complementary site for the seed region of miR‐7‐5p. Next, luciferase reporter assay was performed to confirm this result. As demonstrated in Fig. 5B,C, the ectopic expression of miR‐7‐5p significantly decreased the expression level of luciferase with WT PAK2 while that with MUT PAK2 was unaffected in either A549 or H1299 cells (*P* < 0.01). Using quantitative real‐time PCR (Fig. 5D) and western blot (Fig. 5E), we further confirmed the mRNA and protein expression levels of PAK4 were significantly decreased in both A549 (*P* < 0.001) and H1299 (*P* < 0.01) cells after mimic transfection. Consistently, downregulation of miR‐7‐5p obviously elevated the protein expression of PAK2 in SPC‐A1 cells (Fig. 5F). Taken together, these results confirm that PAK2 is a direct target gene of miR‐7‐5p in NSCLC cells.

### Restoration of PAK2 abolished the effects of miR‐7‐5p on cell proliferation, cell cycle progression, and apoptosis

**Figure 6:**
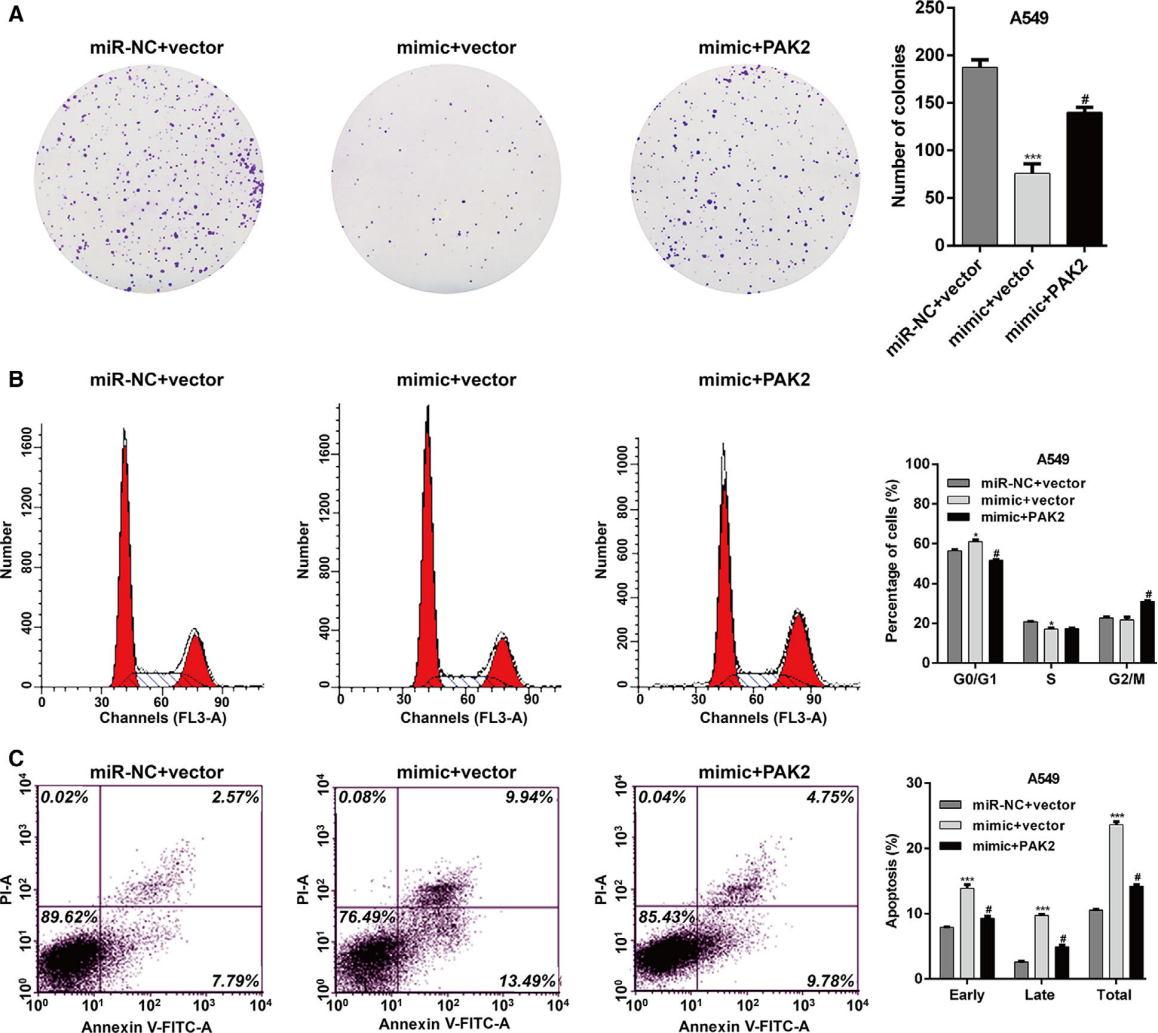
PAK2 can rescue the phenotypic changes caused by miR‐7‐5p. A549 cells were cotransfected with miR‐7‐5p mimic/miR‐NC and with PAK2 overexpression plasmid/empty vector. (A) Cell proliferation, (B) cell cycle distribution, and (C) apoptosis were determined by colony formation and flow cytometry analysis. The data were statistically analyzed by one‐way analysis of variance for comparing multiple groups. Data are shown as the means ± SD of three independent experiments. *P < 0.05, ***P < 0.001 vs. miR‐NC + vector; #*P* < 0.05 vs. mimic + vector.

To further explore whether PAK2 was a downstream regulator in miR‐7‐5p‐mediated cellular function, rescue experiments were performed by constructing an overexpression plasmid of PAK2 (pcDNA3.1‐PAK2) to transfect into A549 cells treated with mimic, followed by colony formation assay, cell cycle, and apoptosis analysis. As expected, the inhibitory effects on cell proliferation (Fig. 6A) and the induced cell cycle G0/G1 arrest (Fig. 6B) and apoptosis (Fig. 6C) caused by mimic were partially abolished by overexpressing PAK2 in A549 cells. These results demonstrated that PAK2 could rescue the phenotypic changes caused by miR‐7‐5p in human NSCLC cells.

## Discussion

miRNAs are known to play critical roles in the development and maintenance of normal cellular events, but aberrant expression of miRNAs may affect the initiation and progression of different cancers 22. In this study, we focused on the clinical significance and biological function of miR‐7‐5p in NSCLC cells. We first found that miR‐7‐5p was significantly downregulated in NSCLC tissues and cell lines. Multivariate analysis indicated that miR‐7‐5p acts as an independent prognostic factor for survival. To our best knowledge, this is the first report to reveal the correlation between miR‐7‐5p expression profiles and clinical factor in NSCLC. Similarly, Suto et al. 23 found that low miR‐7 expression was an independent prognostic factor for poor survival in colorectal cancer. In addition, miR‐7‐5p has been shown to function as a key tumor suppressor in a variety of tumor types, including breast cancer 10, melanoma 11, glioblastoma 12, and hepatocellular carcinoma 13.

Then, function or loss of function of experiments confirmed that miR‐7‐5p significantly inhibited cell proliferation and induced G0/G1 cell cycle arrest and apoptosis in NSCLC cells. It is known that miRNAs can have numerous targets carrying sequences complementary to the seed sequence in the 5′ end of the miRNA 24. Regulation of PAK2 by miR‐7‐5p was confirmed by *in vitro* transfection experiment in A549 and H1299 and by strongly suggesting the direct binding of the miR‐7‐5p 5′ seed to the PAK2 3’‐UTR with the use of dual‐luciferase reporter assay. Overexpression or amplification of PAK2 has been shown in gastric cancer and melanoma 19, 20. Some reports also revealed that PAK2 expression was closely associated with tumor malignancy and clinical outcome, which indicates that PAK2 may contribute to disease development and progression 20. Furthermore, we found that the effect of miR‐7‐5p on NSCLC cell proliferation, cell cycle progression, and apoptosis could partially be reversed by PAK2 overexpression. These data suggest that downregulation of PAK2 may not all but at least partially responsible for miR‐7‐5p regulating NSCLC cell proliferation, cell cycle distribution, and apoptosis in NSCLC cells.

Previous studies have pointed that PAKs participate in the cell growth signaling and transformation 25. Members of the MAP kinase cascade such as JNK and p38 could be activated by PAKs 1–3 in related study 26. Moreover, JNK and p38 signaling pathway are considered as critical signal transduction pathways involved in cell proliferation, cell cycle arrest, and apoptosis 27. Besides, PAK has been shown to protect apoptotic signals by promoting translocation of Raf‐1 to the mitochondria, triggering formation of Raf‐1‐Bcl‐2 complexes, and depressing the formation of BAD‐Bcl‐2 complexes 28. Two transcription factors, NF‐κB and Forkhead, which are associated with cell apoptosis, can be stimulated and inhibited by PAK, respectively 29, 30. We thus suggest that downregulation of PAK2 could integrate various signaling pathways that are critical to cell growth and survival, therefore leading to miR‐7‐5p‐mediated inhibition of NSCLC cell proliferation and induction of G0/G1 arrest and apoptosis. However, the molecular mechanisms underlying miR‐7‐5p/PAK2 action in NSCLC still require further investigation.

In conclusion, the present study shows that low miR‐7‐5p expression in NSCLC is associated with advanced clinical stage, large tumor size, and poor outcome. The effect of miR‐7‐5p on inhibition of cell proliferation and induction of G0/G1 arrest and apoptosis is mediated mainly by targeting PAK2. These results suggest that miR‐7‐5p and PAK2 might serve as promising prognostic biomarkers and potential therapeutic targets in NSCLC.

## Conflict of interest

The authors declare no conflict of interest.

## Author contributions

LJS conceived and designed the study. LQ and WXP mainly performed the experiments and gathered the data. GL and SJX analyzed the data and wrote the paper. SJX revised the manuscript. All authors read and approved the final manuscript.
